# Treatment patterns and characteristics of patients with migraine: results from a retrospective database study in Japan

**DOI:** 10.1186/s10194-024-01722-5

**Published:** 2024-02-08

**Authors:** Tsubasa Takizawa, Takahiro Kitano, Masahiro Iijima, Kanae Togo, Naohiro Yonemoto

**Affiliations:** 1 Department of Neurology, Keio University School of Medicine, Tokyo, Japan; 2 Health & Venue, Pfizer Japan Inc., Shinjuku Bunka Quint Building, 3-22-7, Yoyogi, Shibuya-ku Tokyo, 151-8589 Japan; 3 Internal Medicine & Hospital Medical Affairs, Pfizer Japan Inc., Tokyo, Japan

**Keywords:** Acute and preventive treatment, Anti-CGRP mAbs, Medical facilities, Medication-overuse headache, Migraine, Retrospective cohort study

## Abstract

**Background:**

Clinical characteristics and treatment practice of patients with migraine in Japan in real-world setting have not been fully investigated. We conducted a retrospective cohort study using claims database to understand the clinical practice of migraine in recent years and to characterize patients potentially not managed well by current treatment options.

**Methods:**

Our study used data from the large claims database maintained by JMDC Inc. Patients with diagnosis of headache or migraine between January 1, 2018, and July 31, 2022, were defined as the headache cohort, and those with migraine diagnosis and prescription of migraine treatments among the headache cohort were included in the migraine cohort. In the headache cohort, characteristics of medical facilities and status of imaging tests to distinguish secondary headache were examined. Treatment patterns and characteristics of patients potentially not managed well by acute/preventive treatment were described in migraine cohort.

**Results:**

In the headache cohort, 989,514 patients were included with 57.0% females and mean age of 40.3 years; 77.0% patients visited clinics (with ≤ 19 bed capacities) for their primary diagnosis, and 30.3% patients underwent imaging tests (computed tomography and/or magnetic resonance imaging). In the migraine cohort, 165,339 patients were included with 65.0% females and mean age of 38.8 years. In the migraine cohort, 95.6% received acute treatment while 20.8% received preventive treatment. Acetaminophen/non-steroidal anti-inflammatory drugs were most common (54.8%) as the initial prescription for migraine treatment followed by triptan (51.4%). First treatment prescription included preventive treatment in 15.6%, while the proportion increased to 82.2% in the fourth treatment prescription. Among patients with more than 12 months of follow-up, 3.7% had prescription patterns suggestive of risk of medication-overuse headache, and these patients were characterized by a higher percentage of females and a higher prevalence of comorbidities.

**Conclusions:**

This study revealed that approximately one-fifth of the patients with migraine visiting medical facilities use preventive drugs. The presence of potential patients at risk of medication-overuse headache and the role of clinics in migraine treatment were also described.

**Supplementary Information:**

The online version contains supplementary material available at 10.1186/s10194-024-01722-5.

## Introduction

Migraine is the most common multifactorial neurologic disorder which has remained as one of the prominent causes affecting patients’ quality of life and daily functions [1]. It is characterized by incidences of relapse and remittance of extreme headache with variable frequencies affecting both men and women [2]. The estimated lifetime global prevalence of migraine was 17.5% including both genders and 21.0% in females and 11.6% in males based on an in-depth literature review of studies published until 2020 [3]. As per the Global Burden of Disease 2019 study, migraine is the second most common cause of disability globally and the first cause of disability among women younger than 50 years [4]. In Japan, the estimated annual prevalence of migraine ranged between 6.0% and 8.9% [5]. Multiple cross-sectional online surveys conducted in Japan also suggested a substantial burden of migraine with poor quality of life, daily life activity impairment, work disability and decreased work productivity, and unmet needs for acute and preventive treatments [6–8]. A cross-sectional study conducted in ~ 30,000 patients with migraine revealed a substantial economic burden in terms of increased direct costs (1.83-fold increased healthcare resource utilization) and indirect costs (1.82-fold increase, primarily driven by increased presenteeism costs), compared to ~ 1,512 matched non-migraine respondents [9].

The primary aim of the acute migraine treatment is to reduce the frequency, severity, and duration of the migraine attack with the objectives of regaining normal functional ability and overall management with or without minimal side effects [10]. The current acute treatments include treatment with prescription and over-the-counter (OTC/nonprescription) drugs including combination non-steroidal anti-inflammatory drugs (NSAIDs)–acetaminophen, aspirin, and caffeine. Other than OTC drugs, NSAIDs, acetaminophen, triptans, ergotamine, and antiemetics are also used [11]. In addition to acute treatment, prevention of migraine in selected patient population helps reduce the clinical, humanistic, and economic burden of the disease [12]. Preventive (prophylactic) treatment includes use of prescription drugs such as beta-blockers, antidepressants, antiepileptics, and calcium-channel blockers [13]. Additionally, anti–calcitonin gene-related peptide (CGRP) monoclonal antibodies (mAbs) have been approved as preventive treatment options in Japan in 2021 [12, 14, 15].

In a previous cross-sectional population-based online survey of 17,071 individuals with migraine in Japan, the status of acute migraine treatment was assessed, and unmet needs were discussed [8]. Of 14,869 individuals using acute treatment, 48.3% reported poor or very poor effectiveness of treatment. During severe headaches, 37.4% respondents reported severe or very severe impairment of daily life activity and work while 47.6% reported some impairment of working ability or activity. Overall, the study reported that approximately three-fourths of Japanese individuals with migraine have unmet needs despite receiving acute migraine treatment [8]. Medication-overuse headache (MOH) caused by overuse of acute treatments has been another concern in patients with migraine in Japan [16]. However, epidemiological information on MOH in Japan is limited. On the other hand, the use of prophylactic treatment with beta-blockers, calcium-channel blockers, antiepileptics, and antidepressants was associated with a high rate of discontinuation within 2 months of treatment initiation [13, 17]. Despite the introduction of anti-CGRP mAbs as the new preventive treatment option, there is insufficient evidence to determine which patients should receive prophylactic medication in the real-world practice [8, 11, 13, 18].

In this context, more detailed understanding of the medical environment and treatment patterns including the effect of changes in prophylactic treatment options for patients with migraine in the current real-word practice is needed. The characteristics of medical facilities providing migraine care and the characteristics of patients who are potentially not managed well with prevailing acute and preventive treatment options or who are at risk of MOH are not well documented till date. To address this evidence gap, we conducted a retrospective noninterventional cohort study in Japan to describe the characteristics of medical facilities treating migraine in Japan, the real-world treatment patterns, and clinical characteristics of patients with migraine, including those who were potentially not managed well by the current acute and/or preventive treatments.

## Methods

### Study design and data source

This was a retrospective database analysis that utilized anonymized claims data from JMDC Inc. JMDC is a large claims database in Japan that collects claims data from health insurance providers for company employees and their dependents. As of December 2023, the database had information on approximately 17 million people [19]. The JMDC database includes diagnosed disease names, coded according to Japanese Claims Codes and International Classification of Diseases 10th revision (ICD10) coding scheme, and details of prescriptions. The JMDC data include data from inpatients, outpatients, and pharmacy claims. Within the JMDC database, the patients can be followed even if they visited multiple medical facilities unless they withdrew from the health insurance. The study period was from January 1, 2018, to July 31, 2022.

### Patient identification

Two patient cohorts were created for the study: 1) headache cohort and 2) migraine cohort. The headache cohort was set to characterize medical facilities treating headache and the status of imaging tests to distinguish secondary headaches in Japan. On the other hand, the migraine cohort was set to characterize the clinical characteristics of patients with migraine in Japan, explore the actual treatment pattern for migraine patients, and explore the characteristics of patients who are potentially not managed well by existing migraine treatments including patients at risk of MOH. Patients (aged ≥ 18 years) with a diagnosis of headache (R51) or migraine (G43) according to the ICD10 between January 1, 2018, and July 31, 2022, with at least 6 months of baseline period were included in the headache cohort. From these, patients with a diagnosis of migraine (G43) were included in the migraine cohort only if they had a prescription for any migraine treatment. Patients were excluded from the migraine cohort if they had a confirmed diagnosis of cluster headache (ICD10: G44.0). Index date for the headache cohort was the first day of the month when a headache or migraine was initially diagnosed in the patient, including a suspicious diagnosis. Index date for the migraine cohort was the day of the first prescription for the migraine treatment. Maximum follow-up duration for both cohorts was up to the date of the last visit from the index date. Patients with at least 12 months of follow-up (12 M-F/U population) were identified in the migraine cohort and results are reported.

### Study variables

The study variables in the headache cohort included patient characteristics such as age at the index date, sex, follow-up durations, diagnosis at index date (headache or migraine), comorbidities during baseline period, characteristics of the medical facilities where index diagnosis was made (number of beds), and the status of imaging tests (computed tomography [CT] and magnetic resonance imaging [MRI]) performed possibly to eliminate secondary headaches and performed in the 3 months before and after the initial diagnosis. Medical institutions were classified as (1) hospitals having 20 or more bed capacities (HPs) or (2) clinics having 19 or less bed capacities (CPs) based on the Medical Care Act [20].

In the migraine cohort, prescriptions for acute and preventive migraine treatment, treatment prescriptions, and prescription period (time from the date of the first prescription to the date when prescription days have elapsed; if another refill for the same medication was observed within 60 days of exhausting the prescription days for the prior prescription, it was regarded as a continuous prescription) were assessed. Treatment patterns included treatments prescribed (type [acute/preventive] and treatment prescriptions [from first to fourth prescriptions of migraine treatment]). The drugs prescribed for acute treatment included acetaminophen or NSAIDs, triptans, and ergotamine and those for preventive treatment included anti-CGRP mAbs, antiepileptics, antidepressants, beta-blockers, and calcium-channel blockers which are approved for treating migraine in Japan.

Using the typical treatment prescriptions, patients who were potentially not managed well by both the acute treatment and the combination of acute and preventive treatments were identified. Patients potentially not managed well by the acute treatment were categorized into two groups: Case 1 and Case 2. Case 1 included patients with at least one triptan switch, and Case 2 included patients with prescription patterns defined as having potential risk of MOH based on the definition as per the International Classification of Headache Disorders, 3rd edition. Moreover, patients potentially not managed well by the combination of acute and preventive treatments were further categorized as Case 3 (no decrease in average acute care drug prescription after introduction of preventive care drugs). More details on all the above-mentioned cases are found in the supplementary material. Characteristics of Cases 1, 2, and 3 were analyzed in patients of the 12 M-F/U population.

### Statistical analysis

Data were analyzed descriptively. Patient characteristics were summarized for the headache cohort and the migraine cohort. A subgroup analysis for imaging tests in the migraine cohort was performed for two subgroups of HPs/CPs. Continuous variables were summarized using mean ± standard deviation (SD) and categorical variables were reported using frequency and proportion. Statistical analyses were performed using the SAS 9.4 (SAS Institute, Cary, NC, USA).

### Ethical considerations

The study protocol was approved by the Institutional Review Board held by MINS (a specified nonprofit organization) on April 5, 2023. It was based on anonymized personal information following privacy laws, and obtaining informed consent from patients was not required. The principles outlined in the Declaration of Helsinki were followed.

## Results

### Patients and baseline characteristics

**Fig. 1:**
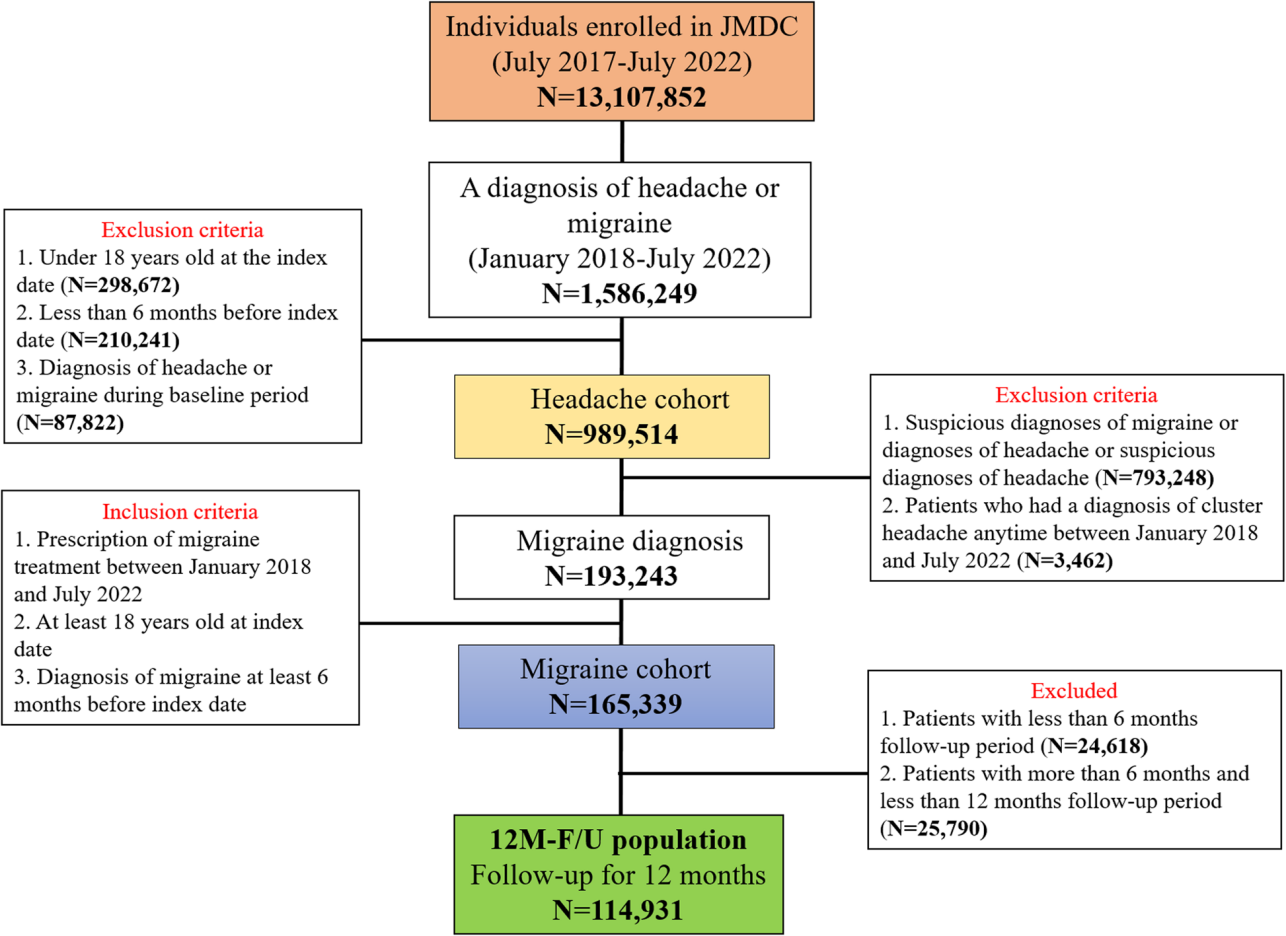
Patient flow and creation of cohorts. 12 M-F/U, 12 months of follow-up

Of the 13,107,852 individuals enrolled in JMDC during the study period, 1,586,249 were diagnosed with either headache or migraine. As per inclusion criteria, a total of 989,514 patients were included in the headache cohort and 165,339 in the migraine cohort (Fig. 1). Regarding the number of patients prescribed with any migraine medication and the new users in the migraine cohort, the proportion of new users has decreased year by year (Supplementary Table 1).

### Baseline characteristics

The mean ± SD age of patients was 40.3 ± 12.9 years and 38.8 ± 11.7 years, 56.6% and 65.0% were female, and the follow-up duration was 23.8 ± 16.3 months and 23.3 ± 15.9 months in the headache and the migraine cohorts, respectively. Of 989,514 patients in the headache cohort, 85.2% patients had index diagnosis of headache. In the headache and migraine cohorts, 32.2% and 34.0% patients had ≥ 1 comorbidities, respectively. The most common were cardiovascular diseases (16.4% and 13.8%) and neurotic, stress-related, and somatoform disorders (10.1% and 13.7%) in the headache and the migraine cohorts, respectively (Table 1).

**Table 1:** Patient demographics and baseline characteristics

	**Headache cohort** *N* = 989,514	**Migraine cohort** *N* = 165,339
*Age at the index date (years)*		
Mean ± SD	40.3 ± 12.9	38.8 ± 11.7
*Sex, n (%)*		
Male	429,089 (43.4)	57,877 (35.0)
Female	560,425 (56.6)	107,462 (65.0)
*Follow-up duration (months)*		
Mean ± SD	23.8 ± 16.3	23.3 ± 15.9
*Diagnosis at index, n (%)*		
Headache	842,630 (85.2)	NA
Migraine	163,559 (16.5)	165,339 (100.0)
*Comorbidities during baseline period, n (%)*		
Total patients with any kind of comorbidities	318,622 (32.2)	56,235 (34.0)
Cerebrovascular disease	36,866 (3.7)	6,103 (3.7)
Hypertension	126,754 (12.8)	15,723 (9.5)
Ischemic heart diseases	21,916 (2.2)	2,671 (1.6)
Peripheral vascular disease	20,813 (2.1)	3,119 (1.9)
Any of the cardiovascular-related comorbidities listed above	162,130 (16.4)	22,763 (13.8)
Malignant neoplasm of brain	476 (0.0)	49 (0.0)
Malignant neoplasms (except for brain)	24,080 (2.4)	3,138 (1.9)
Meningitis	976 (0.1)	176 (0.1)
Mood (affective) disorders	69,504 (7.0)	17,154 (10.4)
Neurotic, stress-related, and somatoform disorders	99,607 (10.1)	22,726 (13.7)
Epilepsy	14,470 (1.5)	3,352 (2.0)
Disorders of thyroid gland	41,440 (4.2)	7,350 (4.4)
Diabetes mellitus	69,989 (7.1)	8,657 (5.2)

*NA* Not applicable, *SD* Standard deviation

### Characteristics of medical facilities and imaging tests

Of 989,514 patients in the headache cohort, 77.0% (761,702) of patients had an index diagnosis of headache or migraine at CPs (Table 2).

**Table 2:** Characteristics of the medical facility and imaging tests in the headache cohort

	**Total patients ** ***N* = 989,514**
*Medical facility visited by patients, n (%)*	
CP (0–19 beds)	761,702 (77.0)
HP (20–99 beds)	46,431 (4.7)
HP (100–199 beds)	56,793 (5.7)
HP (200–299 beds)	27,197 (2.7)
HP (300–499 beds)	60,194 (6.1)
HP (≥ 500 beds)	48,627 (4.9)
*Imaging test (CT and/or MRI) undergone by patients, n (%)*	
Total	299,707 (30.3)
At CP	153,734 (20.2)^a^
At HP	172,856 (72.7)^b^

*CP* Clinics having 19 or less bed capacities, *CT* Computed tomography, *HP* Hospitals having 20 or more bed capacities, *MRI* Magnetic resonance imaging

^a^Calculated % using 761,702 as denominator value

^b^Calculated % using 237,772 as denominator value

A total of 30.3% (299,703) patients among the headache cohort underwent imaging tests (CT and/or MRI). Amongst 237,772 patients who consulted HPs, 172,856 (72.7%) underwent imaging tests. Amongst 761,702 patients who consulted CPs, 153,734 (20.2%) underwent imaging tests (Table 2).

### Treatment patterns

During the entire study period, of the 165,339 patients from the migraine cohort, 158,098 (95.6%) received acute treatment while 34,309 (20.8%) received preventive treatment; specifically, 131,030 (79.2%) received acute treatment alone, 7,241 (4.4%) received preventive treatment alone, and 27,068 (16.4%) received both acute and preventive treatments (Supplementary Table 1).

**Fig. 2:**
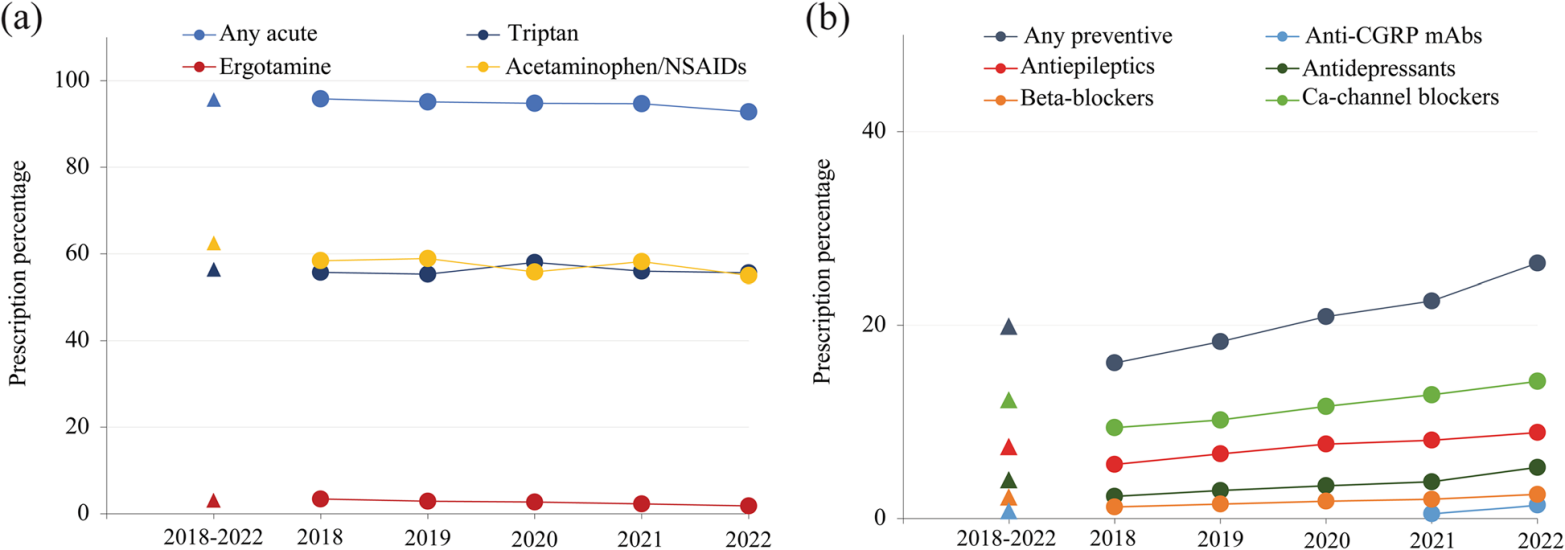
Treatment patterns in patients in the migraine cohort from 2018 to 2022. **a** Trend of acute treatment over years. **b** Trend of preventive treatment over years. Number of patients: 2018-2022 (n=165,339), 2018 (n=35,614), 2019 (n=44,253), 2020 (n=47,719), 2021 (n=61,949), 2022 (n=44,307). Ca, calcium; CGRP, calcitonin gene-related peptide; mAb, monoclonal antibody; NSAID, non-steroidal anti-inflammatory drug

Among the acute treatments, 103,004 (62.3%) patients received acetaminophen and other NSAIDs, 92,543 (56.0%) received triptans, and only 4,717 (2.9%) received ergotamine and caffeine (Supplementary Table 1). From the acetaminophen and NSAIDs class, most patients were prescribed loxoprofen (61,939; 37.5%) and acetaminophen (48,087; 29.1%) while those who were prescribed triptans had rizatriptan (31,097; 18.8%), sumatriptan (28,319; 17.1%), and eletriptan (24,325; 14.7%) in their prescriptions. Of the patients who received preventive treatment, most received calcium-channel blockers (20,451; 12.4%), mostly lomerizine (19,714; 11.9%), while very few received anti-CGRP mAbs (675; 0.4%) (Supplementary Table 1). From the year 2018 to 2022, the use of preventive treatment increased from 16.1% to 26.4%. The use of acute treatment (95.8% in 2018, and 92.8% in 2022) remained generally stable and high (Fig. 2a and b).

### Treatment prescriptions

The number of patients was the highest for the first prescription (165,339) group and was the lowest for the fourth prescription (1,227) group. The use of acute treatment was reduced from 94.3% (155,890/165,339) at the first prescription to 19.0% (233/1,227) at the fourth prescription. Moreover, the use of triptans reduced from 51.4% (85,023/165,339) at the first prescription to 3.9% (48/1,227) at the fourth prescription and acetaminophen and NSAIDs reduced from 54.8% (90,605/165,339) to 12.7% (156/1,227) (Table 3). Noteworthily, preventive treatment was increased from 15.6% (25,801/165,339) at the first prescription to 82.2% (1,009/1,227) at the fourth prescription. Use of anti-CGRP mAbs as preventive treatment was increased from ~ 0.0% (29/165,339) at the first prescription to 14.5% (178/1,227) at the fourth prescription. Similarly, the use of antiepileptics (4.7% to 23.1%), antidepressants (2.0% to 20.4%), beta-blockers (1.1% to 10.9%), and calcium-channel blockers (8.8% to 14.8%) was also notably increased from the first to fourth treatment prescription (Table 3).

**Table 3:** Treatment prescriptions in the migraine cohort

	**First prescription (*N* = 165,339)**		**Second prescription (*N* = 26,537)**		**Third prescription (*N* = 4,937)**		**Fourth prescription (*N* = 1,227)**	
	**n**	**(%)**	**n**	**(%)**	**n**	**(%)**	**n**	**(%)**
*Any acute treatment*	155,890	94.3	18,540	69.9	1,682	34.1	233	19.0
Acetaminophen and/or NSAIDs	90,605	54.8	11,114	41.9	1,090	22.1	156	12.7
Acetaminophen and/or NSAIDs only	64,018	38.7	10,375	39.1	1,022	20.7	150	12.2
+ Triptan	20,211	12.2	168	0.6	9	0.2	1	0.1
+ Ergotamine	784	0.5	27	0.1	4	0.1	0	0.0
+ Preventive treatment	3,073	1.9	500	1.9	52	1.1	5	0.4
+ Others	2,519	1.5	44	0.2	3	0.1	0	0.0
Triptan	85,023	51.4	6,998	26.4	468	9.5	48	3.9
Triptan only	51,674	31.3	5,946	22.4	408	8.3	44	3.6
+ Acetaminophen and/or NSAIDs^a^	20,211	12.2	168	0.6	9	0.2	1	0.1
+ Ergotamine	187	0.1	11	0.0	1	0.0	0	0.0
+ Preventive treatment	9,747	5.9	802	3.0	49	1.0	2	0.2
+ Others	23,415	14.2	239	0.9	10	0.2	2	0.2
Ergotamine	3,887	2.4	659	2.5	138	2.8	30	2.4
Ergotamine only	2,622	1.6	565	2.1	119	2.4	23	1.9
+ Acetaminophen and/or NSAIDs^a^	784	0.5	27	0.1	4	0.1	0	0.0
+ Triptan^a^	187	0.1	11	0.0	1	0.0	0	0.0
+ Preventive treatment	185	0.1	53	0.2	14	0.3	5	0.4
+ Others	1,080	0.7	41	0.2	5	0.1	2	0.2
*Any preventive treatment*	25,801	15.6	9,445	35.6	3,374	68.3	1,009	82.2
Anti-CGRP mAb	29	0.0	155	0.6	211	4.3	178	14.5
Anti-CGRP mAb only	18	0.0	151	0.6	206	4.2	174	14.2
+ Acetaminophen and/or NSAIDs	1	0.0	1	0.0	1	0.0	0	0.0
+ Triptan	2	0.0	1	0.0	1	0.0	0	0.0
+ Ergotamine	0	0.0	0	0.0	0	0.0	0	0.0
+ Other preventive treatment	2	0.0	2	0.0	3	0.1	2	0.2
+ Others	6	0.0	0	0.0	0	0.0	2	0.2
Antiepileptics	7,832	4.7	2,793	10.5	1,068	21.6	284	23.1
Antiepileptics only	3,033	1.8	2,193	8.3	963	19.5	273	22.2
+ Acetaminophen and/or NSAIDs	908	0.5	132	0.5	20	0.4	1	0.1
+ Triptan	2,265	1.4	185	0.7	18	0.4	0	0.0
+ Ergotamine	44	0.0	12	0.0	6	0.1	0	0.0
+ Other preventive treatment	331	0.2	206	0.8	54	1.1	7	0.6
+ Others	1,251	0.8	65	0.2	7	0.1	3	0.2
Antidepressants	3,386	2.0	1,632	6.1	715	14.5	250	20.4
Antidepressants only	771	0.5	1,189	4.5	631	12.8	234	19.1
+ Acetaminophen and/or NSAIDs	311	0.2	96	0.4	9	0.2	1	0.1
+ Triptan	1,095	0.7	96	0.4	11	0.2	1	0.1
+ Ergotamine	24	0.0	8	0.0	3	0.1	1	0.1
+ Other preventive treatment	307	0.2	191	0.7	54	1.1	12	1.0
+ Others	878	0.5	52	0.2	7	0.1	1	0.1
Beta-blockers	1,804	1.1	671	2.5	361	7.3	134	10.9
Beta-blockers only	906	0.5	539	2.0	331	6.7	128	10.4
+ Acetaminophen and/or NSAIDs	181	0.1	43	0.2	12	0.2	0	0.0
+ Triptan	370	0.2	26	0.1	4	0.1	1	0.1
+ Ergotamine	11	0.0	4	0.0	3	0.1	1	0.1
+ Other preventive treatment	69	0.0	42	0.2	10	0.2	4	0.3
+ Others	267	0.2	17	0.1	1	0.0	0	0.0
Ca-channel blockers	14,511	8.8	4,616	17.4	1,114	22.6	182	14.8
Ca-channel blockers only	4,187	2.5	3,585	13.5	1,037	21.0	169	13.8
+ Acetaminophen and/or NSAIDs	1,672	1.0	228	0.9	10	0.2	3	0.2
+ Triptan	6,015	3.6	494	1.9	15	0.3	0	0.0
+ Ergotamine	106	0.1	29	0.1	2	0.0	3	0.2
+ Other preventive treatment	325	0.2	223	0.8	45	0.9	7	0.6
+ Others	2,206	1.3	57	0.2	5	0.1	0	0.0

*Ca* Calcium, *CGRP* Calcitonin gene-related peptide, *mAb* Monoclonal antibody, *NSAID* Non-steroidal anti-inflammatory drug

^a^Same results shown as Acetaminophen and/or NSAIDs + Triptan, Acetaminophen and/or NSAIDs + Ergotamine, and Triptan + Ergotamine

In the migraine cohort, 38.7% (64,018) patients started their migraine treatment with only acetaminophen and/or NSAIDs without any other migraine drugs given in combination, followed by acetaminophen and/or NSAIDs in combination with triptan (12.2%, 20,211) and triptan in combination with preventive drugs (5.9%, 9,747) (Table 3). In contrast, of 1,227 patients receiving the fourth treatment prescription, only 12.2% (150) patients received acetaminophen and/or NSAIDs without any other migraine drugs, while prescriptions for only anti-CGRP mAbs (14.2%, 174), only antiepileptics (22.2%, 273), only antidepressants (19.1%, 234), and only calcium-channel blockers (13.8%, 169) were more common (Table 3). Further, in the fourth treatment prescription, few patients received combination therapies. Specifically, preventive drugs in combination with antiepileptics (0.6%, 7), antidepressants (1.0%, 12), and calcium channel blockers (0.6%, 7).

Prescription periods (median [25th-75th percentiles]) for preventive drugs were 95 days [31-195] for anti-CGRP mAbs, 48 days [20-159] for antiepileptics, 44 days [21-143] for antidepressants, 37 days [14-126] for beta-blockers, and 30 days [14-97] for calcium-channel blockers. (Data not shown).

### Characteristics of patients potentially not managed well by acute/preventive treatment

Table 4 presents data from the 12 M-F/U population; the mean ± SD age was 39.2 ± 11.4 years and 64.4% were females. The characteristics of patients potentially not managed well by acute treatment (Case 1 triptan switch, 1.2%; Case 2 MOH, 3.7%) or combination of acute and preventive treatments (Case 3-1 conventional prevention, 0.3%; Case 3-2 anti-CGRP mAbs, 0.03%) are also shown. For Case 1, the mean ± SD age was 37.4 ± 10.3 years and 73.4% were females. For Case 2, the mean ± SD age was 42.3 ± 10.5 years and 70.2% were females.

**Table 4:** Characteristics of patients potentially not managed well by acute, preventive, or combination treatment

	**Total** **(12 M-F/U population)**		**Triptan switch** **Case 1**		**MOH** **Case 2**		**No decrease of acute medication with conventional preventive treatment** **Case 3-1**^**a**^		**No decrease of acute medication with Anti-CGRP mAbs treatment** **Case 3-2**^**a**^	
	***N* = 114,931**		***N* = 1,337**		***N* = 4,229**		***N* = 651**		***N* = 62**	
**Percentage** **(*N*/Population 2)**	**100%** **(114,931/114,931)**		**1.2%** **(1,337/114,931)**		**3.7%** **(4,229/114,931)**		**23.0%** **(651/2,832**^**b**^**)**		**29.0%** **(62/214**^**c**^**)**	
*Age at the index date (years)*										
Mean ± SD	39.2 ± 11.4		37.4 ± 10.3		42.3 ± 10.5		40.2 ± 10.2		37.5 ± 8.5	
*Sex*	n	%	n	%	n	%	n	%	n	%
Male	40,935	35.6	356	26.6	1,259	29.8	212	32.6	17	27.4
Female	73,996	64.4	981	73.4	2,970	70.2	439	67.4	45	72.6
*Comorbidities during baseline period*	n	%	n	%	n	%	n	%	n	%
Total patients	38,044	33.1	396	29.6	2,044	48.3	244	37.5	23	37.1
Cerebrovascular disease	4,061	3.5	32	2.4	194	4.6	32	4.9	2	3.2
Hypertension	10,607	9.2	68	5.1	656	15.5	58	8.9	2	3.2
Ischemic heart diseases	1,838	1.6	13	1.0	106	2.5	11	1.7	1	1.6
Peripheral vascular disease	2,145	1.9	22	1.6	115	2.7	13	2.0	1	1.6
Any of the cardiovascular-related comorbidities listed above	15,453	13.4	121	9.1	871	20.6	96	14.7	5	8.1
Mood (affective) disorders	11,101	9.7	128	9.6	738	17.5	94	14.4	10	16.1
Neurotic, stress-related, and somatoform disorders	15,126	13.2	171	12.8	918	21.7	110	16.9	9	14.5

*CGRP* Calcitonin gene-related peptide, *12 M-F/U* 12 months of follow-up, *mAb* Monoclonal antibody, *MOH* Medication-overuse headache, *SD* Standard deviation

^a^No decrease in average acute care drug prescription for 3 months after the start of preventive treatment

^b^The number of patients who continued conventional preventive treatment at least 3 months

^c^The number of patients who continued anti-CGRP mAb treatment at least 3 months

Patients at risk of MOH (Case 2) had higher proportion of females (70.2%) compared to the 12 M-F/U population (64.4%). They also had the highest proportion of comorbidities (48.3%); major were cardiovascular (20.6%) and neurotic or stress related (21.7%). The proportion of patients receiving preventive treatment anytime during the follow-up period was 33.9% (1,434) among patients at risk of MOH, while that among the 12 M-F/U population was 20.6% (23,623).

For Case 3-1, the mean ± SD age of patients was 40.2 ± 10.2 years; 67.4% were females. For Case 3-2, the mean ± SD age of patients was 37.5 ± 8.5 years; 72.6% were females. The most prevalent comorbidities in patients of Case 3-1 and Case 3-2 were cardiovascular, mood disorders, and neurotic, stress-related, and somatoform disorders.

## Discussion

Our study had four major findings. First, we found that the majority of patients with migraine received their first medical care from CPs and imaging tests were more likely to be performed at HPs. Second, the proportion of prescriptions for prophylactic drugs increased as treatment progressed from the first to fourth prescription, though number of patients requiring treatment decreased. Third, 3.7% patients were found to have prescribing patterns that put them at risk of MOH caused by inappropriate use of acute treatments, and these patients were characterized by a higher percentage of females than migraine patients overall and a higher prevalence of comorbidities such as mood disorders and neurotic, stress-related, and somatoform disorders. Finally, the prescribing patterns showed that while acute treatment remained the primary choice for migraine, the percentage of prescriptions for prophylaxis has been rising between 2018 and 2022.

The finding of the higher prevalence in females as compared to males in this study is in line with the results of previous studies conducted in Japan, the United States of America (USA), and Italy [21–23]. The mean age of diagnosed patients in the migraine cohort was 38.8 years which was consistent with previous findings [24]. Our study suggested that patients with headache consulted CPs rather than HPs as evident by the results of the characteristics of medical facilities. The reasons could be that the symptoms were considered as nonfatal [24, 25].

In the headache cohort, when data on imaging tests 3 months before and after diagnosis were analyzed, ~ 1/3 (30.3%) of patients had undergone imaging tests and the proportion of patients advised imaging tests (CT/MRI) was higher for HPs (72.7%) compared to the proportion for CPs (20.2%). This finding is in line with a previous Japanese epidemiological study [23]. As per standard of care, imaging tests are recommended to evaluate if headaches are secondary to other causes such as brain tumor [11]. Recommendations of imaging tests for distinguishing the diagnosis of primary headache like migraine or secondary headache also contribute to an increase in the economic burden on patients worldwide due to their high costs, as evident from previous epidemiological studies [5–7, 9, 14, 24, 26]. However, the proportion of imaging tests estimated in this study may not be a true representation of clinical practice in Japan and would probably be an underestimation because the status of imaging tests was explored among patients with diagnosis of headache which likely includes various health conditions in claims databases.

Acetaminophen or NSAIDs (62.3%) remained the first choice for the most prescribed drugs and triptans (56.0%) were the second choice for acute treatment of migraine in Japan in the present study. This is consistent with a previous report wherein 53.7% (525/977) of patients were prescribed triptans as acute treatment for episodic and chronic migraine treatment in Japan [18]. These results are comparable with the results of the study conducted in the USA which showed that 47.9% (112/234) patients with migraine were prescribed acetaminophen while 44.9% (105/234) were prescribed triptans [27]. The higher usage of these drugs in our study was observed as compared to that in a previous study [23] because the previous study included the patients who took only OTC drugs, whereas our study included patients who received drug treatment in medical facilities. Although acute treatment remained the most preferred choice for treatment of migraine [18], major limitations in patients uncontrolled by acute treatment were observed, including dissatisfaction from patients, multiple drugs in a single prescription, numerous side effects, and risk of MOH [17]. We also observed a steady increase in the percentage of patients being prescribed preventive treatment over a period of 5 years from 2018 (16.1%) to 2022 (26.4%). The previous Japanese report showed that prescriptions of preventive drugs comprised mainly of antiepileptics and calcium-channel blockers in 2014 [18]. In the present study, it was found that calcium-channel blockers were the most commonly prescribed preventive medication (12.4%), followed by antiepileptics (7.3%), antidepressants (3.7%), beta-blockers (1.8%), and anti-CGRP mAbs (0.4%). A recent survey-based observational study conducted in Germany and Spain showed that of 20,756 patients, 22.7% received migraine prescription comprised of at least one preventive drug [28]. This further supports our results on preventive treatment prescriptions. Anti-CGRP mAbs, approved in Japan in 2021, have also been increasingly prescribed for the treatment of migraine from 2021 to 2022. Another reason for the increase in preventive treatment could be the rising awareness about preventive treatment by the recommendation of the revised clinical guideline for headache especially after approval of anti-CGRP mAbs in 2021. Although the study population included a relatively small number of new patients in later years, it might affect the higher percentage of preventive treatment to some extent; the effect would be limited because most patients discontinued preventive treatment within half a year.

Furthermore, most patients (94.3%) received acute treatment while only a small proportion of patients (15.6%) received preventive treatment in the first treatment prescription. However, as the treatment progressed from first prescription to fourth prescription, the emphasis on acute treatment decreased and that on prevention increased. This result should be interpreted in the light of the fact that the number of patients receiving treatment decreased as treatment progressed from the first to fourth prescription. However, the increasing trend toward later prescription was consistent with the national clinical practice guideline of headache [29], which states that prophylactic therapy should be offered to patients whose symptoms cannot be controlled with acute treatment, and we consider that the result was likely to reflect the actual treatment practice.

We have also described the characteristics of patients potentially not managed well by acute treatment. In our study, we observed that 3.7% patients in the migraine cohort were at risk of MOH. Previously, it was reported that MOH affects 1–2% of the general population [30]. It should be noted that MOH prevalence estimated in this study is in patients with migraine and it may be underestimated as OTC drug use is not included in the JMDC database. Among the 12 M-F/U population, around one-third (33.1%) had comorbidities, while amongst those at risk of MOH, around half (48.3%) had comorbidities; the most common comorbidities in both these groups were neurotic and stress-related disorders and cardiovascular disorders. Thus, patients at risk of MOH had a substantially higher proportion of comorbidities which is also corroborated by a previous report [31]. Patients at risk of MOH had higher percentage of females and higher mean age compared to the 12 M-F/U population, which was consistent with previous findings [31].

We also showed that there may be patients who were not able to decrease acute treatment despite the introduction of preventive treatment as Cases 3-1 and 3-2. The number of patients in Cases 3-1 and 3-2 looks relatively small, the reason could be the definition of the cases which required patients with continuous use of preventive treatment for 3 months to be included in Cases 3-1 and 3-2, which may have failed to include patients who used preventive treatment at home or whose treatment was prescribed quarterly.

Given these limitations of acute treatments, some patients would be benefitted by use of preventive treatment options. As our study showed, the use of preventive treatment has been rising. In recent years, preventive treatments are gaining popularity and are being increasingly used in carefully selected groups of patients as reported in previous studies [13, 18]. The American Headache Society does not consider calcium-channel blockers as an option for preventive treatment of migraine in the USA [32], while they are rarely recommended as preventive medication in Canada [33]. In contrast, the calcium-channel blockers are highly prescribed preventive drugs in Japan as recommended by the Japanese Society of Neurology [11]. Although they remain the preferred choice as prophylactic medication for migraine in Japan [13], an increase in the prescriptions of anti-CGRP mAbs has been seen after their approval in Japan in 2021. Since the anti-CGRP mAbs were introduced recently and guidelines for appropriate use of these drugs are limiting the qualified physicians who can prescribe anti-CGRP mAbs, use of the drug may increase in the future. As to MOH, it is considered to be preventable and treatable, and treatment options include discontinuation of acute medications and prescription of prophylactic medications [34–37]. The potential MOH risk by overuse of acute treatment has been observed especially in patients with comorbidities such as mood disorders and neurotic, stress-related, and somatoform disorders. The appropriate use of preventive treatment may reduce this risk. Our study revealed that around one-fifth of the patients (20.6%), who were followed up for 12 months, received prescriptions of preventive treatment. The proportion of patients who received preventive treatment among MOH patients (33.9%) was higher than that of the whole migraine patients, which is quite higher than a previous finding [16]. This may indicate that further awareness of preventive treatment is still needed to better manage MOH. However, further studies considering the specialty of physicians treating migraine and the severity of migraine are needed in Japanese real-world setting.

This study has some limitations. As the JMDC database is a claims database from health insurance providers for company employees, data for patients aged ≥ 75 years were not available and the data for patients aged ≥ 65 years were very limited. However, this is not a major limitation as migraine is known to be more prevalent in the working age group of people who are covered in our study. Additional limitations of the database study are that OTC drugs were not recorded, and frequency of migraine attacks was not observable. Furthermore, the database does not cover the actual drug use, for example, the prescription for 10 times as needed is recorded as a prescription for 10 days in the database. Although some patients might have used the drugs within 5 days and some used over 10 days, we assumed that the drugs were used within 1 month when assessing the potential risk of MOH. Nevertheless, this large retrospective study provided vital population-based data on the treatment patterns of migraine in the Japanese population. As the severity of migraine was not observable in this study, further studies assessing the real-world treatment patterns of migraine stratified by disease severity are needed.

## Conclusions

Our study showed an inclination of patients towards consulting CPs rather than HPs in the primary diagnosis and treatment of migraine. This study also revealed that approximately one-fifth of the migraine patients use preventative drugs, the proportion of prophylactic prescriptions has been rising over the past 5 years. We also found that patients with treatment patterns that put them at risk of MOH were more likely to be females and with a higher prevalence of comorbidities such as mood disorders and neurotic, stress-related, and somatoform disorders.

### Supplementary Information

**Additional file 1: Supplementary Table 1. **Treatment prescribed in patients with migraine during follow-up in the migraine cohort. 

## Data Availability

The datasets generated and/or analyzed during the current study are not publicly available because the data was obtained from JMDC Inc.

## Abbreviations

Ca: Calcium
CGRP: Calcitonin gene-related peptide
CP: Clinics having 19 or less bed capacities
CT: Computed tomography
EHF: European Headache Federation
HP: Hospitals having 20 or more bed capacities
ICD10: International Classification of Diseases 10th revision
mAb: Monoclonal antibody
MOH: Medication-overuse headache
MRI: Magnetic resonance imaging
NA: Not applicable
no.: Number
NSAID: Non-steroidal anti-inflammatory drug
OTC: Over-the-counter
SD: Standard deviation
12 M-F/U: 12 Months of follow-up

## References

[1] Amiri P, Kazeminasab S, Nejadghaderi SA, Mohammadinasab R, Pourfathi H, Araj-Khodaei M et al (2022) Migraine: a review on its history, global epidemiology, risk factors, and comorbidities. Front Neurol 12:800605. 10.3389/fneur.2021.800605 10.3389/fneur.2021.800605 PMC8904749 35281991

[2] Serrano D, Lipton RB, Scher AI, Reed ML, Stewart WF, Adams AM (2017). Fluctuations in episodic and chronic migraine status over the course of 1 year: implications for diagnosis, treatment and clinical trial design. J Headache Pain.

[3] Stovner LJ, Hagen K, Linde M, Steiner TJ (2022). The global prevalence of headache: an update, with analysis of the influences of methodological factors on prevalence estimates. J Headache Pain.

[4] Steiner TJ, Stovner LJ, Jensen R, Uluduz D, Katsarava Z (2020). Migraine remains second among the world’s causes of disability, and first among young women: findings from GBD2019. J Headache Pain.

[5] Igarashi H, Ueda K, Jung S, Cai Z, Chen Y, Nakamura T (2020). Social burden of people with the migraine diagnosis in Japan: evidence from a population-based cross-sectional survey. BMJ Open.

[6] Matsumori Y, Ueda K, Komori M, Zagar AJ, Kim Y, Jaffe DH et al (2022) Burden of migraine in Japan: results of the ObserVational survey of the Epidemiology, tReatment, and Care Of MigrainE (OVERCOME [Japan]) study. Neurol Ther 11(1):205–222. 10.1007/s40120-021-00305-9 10.1007/s40120-021-00305-9 PMC8857353 34862581

[7] Sakai F, Hirata K, Igarashi H, Takeshima T, Nakayama T, Sano H (2022). A study to investigate the prevalence of headache disorders and migraine among people registered in a health insurance association in Japan. J Headache Pain.

[8] Takeshima T, Ueda K, Komori M, Zagar AJ, Kim Y, Jaffe DH (2022). Potential unmet needs in acute treatment of migraine in Japan: results of the OVERCOME (Japan) study. Adv Ther.

[9] Kikui S, Chen Y, Todaka H, Asao K, Adachi K, Takeshima T (2020). Burden of migraine among Japanese patients: a cross-sectional National Health and Wellness Survey. J Headache Pain.

[10] Khan J, Al Asoom LI, Al Sunni A, Rafique N, Latif R, Saif S et al (2021) Genetics, pathophysiology, diagnosis, treatment, management, and prevention of migraine. Biomed Pharmacother 139. 10.1016/j.biopha.2021.111557 10.1016/j.biopha.2021.111557 34243621

[11] Headache Clinical Practice Guideline Development Committee (2021) Clinical practice guideline for headache disorders 2021. Japanese Society of Neurology, Japanese Headache Society, and Japanese Society of Neurological Therapeutics (eds). Igaku-Shoin, Tokyo

[12] Takizawa T, Ohtani S, Watanabe N, Miyazaki N, Ishizuchi K, Sekiguchi K (2022). Real-world evidence of galcanezumab for migraine treatment in Japan: a retrospective analysis. BMC Neurol.

[13] Meyers JL, Davis KL, Lenz RA, Sakai F, Xue F (2019). Treatment patterns and characteristics of patients with migraine in Japan: a retrospective analysis of health insurance claims data. Cephalalgia.

[14] Davis L, Torphy B (2022). Managing migraine on the frontline: identifying disease, understanding burden, and incorporating CGRP pathway-targeting therapies in primary care. Br J Pain.

[15] Ihara K, Ohtani S, Watanabe N, Takahashi N, Miyazaki N, Ishizuchi K (2023). Predicting response to CGRP-monoclonal antibodies in patients with migraine in Japan: a single-centre retrospective observational study. J Headache Pain.

[16] Katsuki M, Yamagishi C, Matsumori Y, Koh A, Kawamura S, Kashiwagi K et al (2022) Questionnaire-based survey on the prevalence of medication-overuse headache in Japanese one city—Itoigawa study. Neurol Sci 43(6):3811–3822. 10.1007/s10072-021-05831-w 10.1007/s10072-021-05831-w PMC8765819 35043356

[17] Bentivegna E, Onan D, Martelletti P (2023). Unmet needs in preventive treatment of migraine. Neurol Ther.

[18] Ueda K, Ye W, Lombard L, Kuga A, Kim Y, Cotton S (2019). Real-world treatment patterns and patient-reported outcomes in episodic and chronic migraine in Japan: analysis of data from the Adelphi migraine disease specific programme. J Headache Pain.

[19] JMDC (2023) JMDC claims database – JMDC Inc. https://www.jmdc.co.jp/en/jmdc-claims-database/. Accessed 19 Dec 2023

[20] Japanese Law Translation (2018) Medical care act: ensuring the medical care delivery system, Act No. 205 of July 30, 2018. https://www.japaneselawtranslation.go.jp/en/laws/view/4006/en

[21] Allais G, Chiarle G, Sinigaglia S, Airola G, Schiapparelli P, Benedetto C (2020). Gender-related differences in migraine. Neurol Sci.

[22] Burch R, Rizzoli P, Loder E (2018). The prevalence and impact of migraine and severe headache in the United States: figures and trends from Government Health studies. Headache.

[23] Hirata K, Ueda K, Komori M, Zagar AJ, Selzler KJ, Nelson AM et al (2021) Comprehensive population-based survey of migraine in Japan: results of the ObserVational survey of the Epidemiology, tReatment, and Care Of MigrainE (OVERCOME [Japan]) study. Curr Med Res Opin 37(11):1945–1955. 10.1080/03007995.2021.1971179 10.1080/03007995.2021.1971179 34429000

[24] Takeshima T, Wan Q, Zhang Y, Komori M, Stretton S, Rajan N (2019). Prevalence, burden, and clinical management of migraine in China, Japan, and South Korea: a comprehensive review of the literature. J Headache Pain.

[25] Takeshima T, Ishizaki K, Fukuhara Y, Ijiri T, Kusumi M, Wakutani Y (2004). Population-based door-to-door survey of migraine in Japan: the Daisen study. Headache.

[26] Manack AN, Buse DC, Lipton RB (2011). Chronic migraine: epidemiology and disease burden. Curr Pain Headache Rep.

[27] Kawata AK, Shah N, Poon JL, Shaffer S, Sapra S, Wilcox TK et al (2021) Understanding the migraine treatment landscape prior to the introduction of calcitonin gene-related peptide inhibitors: results from the Assessment of TolerabiliTy and effectiveness in migrAINe patients using preventive treatment (ATTAIN) study. Headache 61(3):438–454. 10.1111/head.14053 10.1111/head.14053 PMC8048891 33594686

[28] Pascual J, Panni T, Dell’Agnello G, Gonderten S, Novick D, Evers S (2023). Preventive treatment patterns and treatment satisfaction in migraine: results of the OVERCOME (EU) study. J Headache Pain.

[29] Araki N, Takeshima T, Ando N, Iizuka T, Igarashi H, Ikeda Y (2019). Clinical practice guideline for chronic headache 2013. Neurol Clin Neurosci.

[30] Russell MB (2019). Epidemiology and management of medication-overuse headache in the general population. Neurol Sci.

[31] Schwedt TJ, Buse DC, Argoff CE, Reed ML, Fanning KM, Hussar CR (2021). Medication overuse and headache burden. Neurol Clin Pract.

[32] Jasvinder C (2023) Migraine Headache Guidelines US Headache Consortium Pharmacologic treatment for episodic migraine prevention in adults. https://emedicine.medscape.com/article/1142556-guidelines?form=fpf

[33] Tzankova V, Becker WJ, Chan TLH (2023). Pharmacologic prevention of migraine. CMAJ.

[34] Chiang CC, Schwedt TJ, Wang SJ, Dodick DW (2016). Treatment of medication-overuse headache: a systematic review. Cephalalgia.

[35] Scheffler A, Messel O, Wurthmann S, Nsaka M, Kleinschnitz C, Glas M (2020). Erenumab in highly therapy-refractory migraine patients: first German real-world evidence. J Headache Pain.

[36] Silberstein SD, Cohen JM, Seminerio MJ, Yang R, Ashina S, Katsarava Z (2020). The impact of fremanezumab on medication overuse in patients with chronic migraine: subgroup analysis of the HALO CM study. J Headache Pain.

[37] Tepper SJ, Diener HC, Ashina M, Brandes JL, Friedman DI, Reuter U (2019). Erenumab in chronic migraine with medication overuse. Neurology.

