# Supplementary material for: Treatment patterns and characteristics of patients with migraine: results from a retrospective database study in Japan
Source: J Headache Pain. 2024 Feb 8;25(1):19. doi: 10.1186/s10194-024-01722-5 (PMC10854051; doi:10.1186/s10194-024-01722-5)
Supplement: Supplementary file 1 — Additional file 1: Supplementary Table 1. Treatment prescribed in patients with migraine during follow-up in the migraine cohort. [file 10194_2024_1722_MOESM1_ESM.docx]

**SUPPLEMENTARY MATERIAL**

**Definitions of cases**

Patients potentially not managed well by acute treatment were categorized into two groups.

Case 1: Triptan switch

It was defined as patients with at least one triptan switch observed within 12 months of the first date of migraine medication prescription after the index date.

Case 2: Patient at risk of MOH

The definition of MOH was based on the International Classification of Headache Disorders 3rd edition (Headache Classification Committee 2018). It was defined as a case at risk of triptan-derived MOH if a patient had a treatment pattern satisfying all the following conditions during the follow-up period:

1) Prescriptions of triptan for a total of ≥10 prescription days within a month from a triptan prescription anytime during the follow-up period.

2) ≥30 prescription days (10 days×3 months) of triptan in consecutive 3 months from the triptan prescription in 1).

For ergotamine, acetaminophen, and NSAIDs, the MOH derived from each drug was defined based on prescription days shown in the table below, and used similar rules as triptans except for number of the days.

| **Drug** | **Prescription days per month** |
| --- | --- |
| Ergotamine | ≥10 days per month |
| Acetaminophen | ≥15 days per month |
| NSAIDs | ≥15 days per month |

NSAID, non-steroidal anti-inflammatory drug

It was defined as a case at risk of MOH from multiple drug classes not individually overused if a patient had a treatment pattern satisfying all the following conditions during the follow‑up period:

1) Prescriptions for more than one of triptan, ergotamine, acetaminophen or NSAIDs within

a month from a prescription of any of these drugs anytime during the follow-up period.

2) Total prescription days of each drug in 1) exceeds 10 days. However, each drug

individually does not exceed the prescription days as per above table.

3) Prescriptions for a total of 30 (10×3) or more days of administration within 90 days of

the initial prescription in 1).

Case 3: Patients without a decrease of acute medication after introduction of preventive drugs

Patients whose acute treatment dosages and frequencies did not decrease even after the initiation of preventive treatment were considered as potentially not managed well by the combination of acute and preventive treatments (Case 3). These patients were subcategorized into two groups:

1) Those who received the conventional preventive treatment (Case 3-1).

2) Those who received the anti-CGRP mAbs as preventive treatment (Case 3-2).

Case 3-1 comprised patients who received the conventional preventive treatment for ≥3 months and received acute treatment with one drug before or after the start of preventive treatment, whose prescription was equal to or greater than the average amount of the same drug during 3 months before and after the introduction of preventive treatment.

Case 3-2 comprised patients who received the anti-CGRP mAbs as preventive treatment for ≥3 months and received acute treatment with one drug before or after the start of anti-CGRP mAb treatment, whose prescription was equal to or greater than the average amount of the same drug during 3 months before and after the introduction of anti-CGRP mAbs.

**References**

Headache Classification Committee of the International Headache Society (IHS) The International Classification of Headache Disorders, 3rd edition (2018) Cephalalgia 38(1):1-211. <https://doi.org/10.1177/0333102417738202>

**Supplementary Table 1. Treatment prescribed in patients with migraine during follow-up in the migraine cohort**

| **Treatment pattern** | **Total**  **N=165,339** | | **2018**  **N=35,614**  **(New user: N=35,614)^a^** | | **2019**  **N=44,253**  **(New user: N=34,224)^a^** | | **2020**  **N=47,719**  **(New user: N=32,367)^a^** | | **2021**  **N=61,949**  **(New user: N=40,946)^a^** | | **2022**  **N=44,307**  **(New user: N=22,188)^a^** | |
| --- | --- | --- | --- | --- | --- | --- | --- | --- | --- | --- | --- | --- |
|  | n | % | n | % | n | % | n | % | n | % | n | % |
| **Acute treatment** | 158,098 | 95.6 | 34,115 | 95.8 | 42,066 | 95.1 | 45,241 | 94.8 | 58,642 | 94.7 | 41,135 | 92.8 |
| *Triptan* | 92,543 | 56.0 | 19,841 | 55.7 | 24,456 | 55.3 | 27,688 | 58.0 | 34,712 | 56.0 | 24,630 | 55.6 |
| Eletriptan | 24,325 | 14.7 | 3,925 | 11.0 | 5,623 | 12.7 | 7,066 | 14.8 | 9,487 | 15.3 | 6,917 | 15.6 |
| Sumatriptan | 28,319 | 17.1 | 6,012 | 16.9 | 7,193 | 16.3 | 7,661 | 16.1 | 9,341 | 15.1 | 5,980 | 13.5 |
| Zolmitriptan | 15,114 | 9.1 | 3,464 | 9.7 | 4,012 | 9.1 | 4,222 | 8.8 | 5,060 | 8.2 | 3,451 | 7.8 |
| Naratriptan | 9,996 | 6.0 | 1,974 | 5.5 | 2,321 | 5.2 | 2,800 | 5.9 | 3,653 | 5.9 | 2,637 | 6.0 |
| Rizatriptan | 31,097 | 18.8 | 6,470 | 18.2 | 7,801 | 17.6 | 8,913 | 18.7 | 11,129 | 18.0 | 7,876 | 17.8 |
| *Ergotamine* | 4,717 | 2.9 | 1,218 | 3.4 | 1,284 | 2.9 | 1,307 | 2.7 | 1,421 | 2.3 | 794 | 1.8 |
| Ergotamine, caffeine | 4,717 | 2.9 | 1,218 | 3.4 | 1,284 | 2.9 | 1,307 | 2.7 | 1,421 | 2.3 | 794 | 1.8 |
| *Acetaminophen and NSAIDs* | 103,004 | 62.3 | 20,796 | 58.4 | 26,048 | 58.9 | 26,617 | 55.8 | 36,027 | 58.2 | 24,371 | 55.0 |
| Acetaminophen | 48,087 | 29.1 | 8,137 | 22.8 | 10,548 | 23.8 | 10,700 | 22.4 | 17,196 | 27.8 | 10,362 | 23.4 |
| Aspirin | 190 | 0.1 | 38 | 0.1 | 42 | 0.1 | 56 | 0.1 | 61 | 0.1 | 45 | 0.1 |
| Indomethacin farnesil | 689 | 0.4 | 53 | 0.1 | 135 | 0.3 | 191 | 0.4 | 225 | 0.4 | 170 | 0.4 |
| Diclofenac | 4,398 | 2.7 | 855 | 2.4 | 1,109 | 2.5 | 1,115 | 2.3 | 1,387 | 2.2 | 877 | 2.0 |
| Mefenamic acid | 1,326 | 0.8 | 360 | 1.0 | 327 | 0.7 | 325 | 0.7 | 345 | 0.6 | 223 | 0.5 |
| Loxoprofen | 61,939 | 37.5 | 13,136 | 36.9 | 16,307 | 36.8 | 16,603 | 34.8 | 20,236 | 32.7 | 14,659 | 33.1 |
| **Preventive treatment** | 34,309 | 20.8 | 5,724 | 16.1 | 8,095 | 18.3 | 9,986 | 20.9 | 13,921 | 22.5 | 11,707 | 26.4 |
| *Anti-CGRP mAbs* | 675 | 0.4 | 0 | 0.0 | 0 | 0.0 | 0 | 0.0 | 304 | 0.5 | 623 | 1.4 |
| Galcanezumab | 369 | 0.2 | 0 | 0.0 | 0 | 0.0 | 0 | 0.0 | 202 | 0.3 | 314 | 0.7 |
| Fremanezumab | 232 | 0.1 | 0 | 0.0 | 0 | 0.0 | 0 | 0.0 | 65 | 0.1 | 222 | 0.5 |
| Erenumab | 154 | 0.1 | 0 | 0.0 | 0 | 0.0 | 0 | 0.0 | 49 | 0.1 | 147 | 0.3 |
| *Antiepileptics* | 12,028 | 7.3 | 1,981 | 5.6 | 2,943 | 6.7 | 3,673 | 7.7 | 5,035 | 8.1 | 3,947 | 8.9 |
| Valproic acid | 12,028 | 7.3 | 1,981 | 5.6 | 2,943 | 6.7 | 3,673 | 7.7 | 5,035 | 8.1 | 3,947 | 8.9 |
| *Antidepressants* | 6,051 | 3.7 | 815 | 2.3 | 1,270 | 2.9 | 1,599 | 3.4 | 2,354 | 3.8 | 2,351 | 5.3 |
| Amitriptyline | 6,051 | 3.7 | 815 | 2.3 | 1,270 | 2.9 | 1,599 | 3.4 | 2,354 | 3.8 | 2,351 | 5.3 |
| *Beta-blockers* | 3,024 | 1.8 | 416 | 1.2 | 652 | 1.5 | 841 | 1.8 | 1,220 | 2.0 | 1,089 | 2.5 |
| Propranolol | 3,024 | 1.8 | 416 | 1.2 | 652 | 1.5 | 841 | 1.8 | 1,220 | 2.0 | 1,089 | 2.5 |
| *Ca-channel blockers* | 20,451 | 12.4 | 3,334 | 9.4 | 4,524 | 10.2 | 5,521 | 11.6 | 7,906 | 12.8 | 6,278 | 14.2 |
| Lomerizine | 19,714 | 11.9 | 3,201 | 9.0 | 4,346 | 9.8 | 5,311 | 11.1 | 7,650 | 12.3 | 6,071 | 13.7 |
| Verapamil | 952 | 0.6 | 150 | 0.4 | 212 | 0.5 | 242 | 0.5 | 314 | 0.5 | 248 | 0.6 |
| **Acute treatment only** | 131,030 | 79.2 | 29,890 | 83.9 | 36,158 | 81.7 | 37,733 | 79.1 | 48,028 | 77.5 | 32,600 | 73.6 |
| **Preventive treatment only** | 7,241 | 4.4 | 1,499 | 4.2 | 2,187 | 4.9 | 2,478 | 5.2 | 3,307 | 5.3 | 3,172 | 7.2 |
| **Acute and preventive treatments** | 27,068 | 16.4 | 4,225 | 11.9 | 5,908 | 13.4 | 7,508 | 15.7 | 10,614 | 17.1 | 8,535 | 19.3 |

Ca, calcium; CGRP, calcitonin gene-related peptide; mAb, monoclonal antibody; NSAID, non-steroidal anti-inflammatory drug.

^a^New user represents number of patients with migraine who received their first treatment prescription in a year.
